# The complete chloroplast genome sequence of *Amomum villosum* Lour.

**DOI:** 10.1080/23802359.2020.1721358

**Published:** 2020-02-03

**Authors:** Mengli Ma, Bingyue Lu

**Affiliations:** Key Laboratory for Research and Utilization of Characteristic Biological Resources in Southern Yunnan, College of Life Science and Technology, Honghe University, Mengzi, PR China

**Keywords:** *Amomum villosum*, complete chloroplast genome, phylogenetic analysis

## Abstract

*Amomum villosum* Lour. (Zingiberaceae) is an important edible and medicinal crop. The complete chloroplast (cp) genome of *A. villosum* was determined using Illumina sequencing platform. The size of whole cp genome was 164,069 bp, containing a small single copy (SSC) region of 15,353 bp and a large single copy (LSC) region of 88,798 bp, which were separated by a pair of inverted repeat (IRs) regions (29,959 bp). The *A. villosum* cp genome contained 133 genes, including eight ribosomal RNA genes (4 rRNA species), 38 transfer RNA genes (30 tRNA species) and 87 protein-coding genes (79 PCG species). The overall GC content of *A. villosum* cp genome is 36.05%. To investigate the evolution status of *A. villosum*, as well as Zingiberales, a phylogenetic tree with *A. villosum* and other 21 species was constructed based on their complete chloroplast genomes. Phylogenetic analysis revealed that *A. villosum *was closely related to *Amomum krervanh*.

*Amomum villosum* Lour. belongs to the ginger family of Zingiberaceae, the dry ripe fruit is called “Sharen” in China, is a commonly used traditional Chinese medicine herb but also used as cooking condiments (Wu and Larsen 2000; Doh et al. 2019). In clinical, “Sharen” is widely used to treat digestive diseases such as abdominal pain, vomiting, and dysentery (Chinese Pharmacopoeia Commission 2015). At present, the research on *A. villosum* mainly focuses on the chemical composition and pharmacological effects, while there are few studies on the molecular aspects (Suo et al. 2018; Ao et al. 2019). The complete chloroplast genome will be useful to shed light on the phylogenetic relationships and will be beneficial for DNA molecular studies in genus *Amomum*.

Fresh leaves of *A. villosum* were collected from Jinping County (22°73′99.86″N, 103°21′43.19″E), Yunnan Province, China. The voucher specimen (LBY20190425) was deposited in Herbarium of Honghe University, China. Approximately 5 g of fresh leaves was harvested for chloroplast DNA isolation (McPherson et al. 2013). After DNA isolation, purified cp DNA was used for short-insert libraries construction (Borgstrom et al. 2011), the whole cp genome sequencing was conducted by BIOZERON Co., Ltd. (Shanghai, China) on the Illumina Hiseq 4000 platform. Then we used the software SOAPdenovo2.04 to assemble the complete cp genome of *A. villosum* (Luo et al. 2012) and the genes were annotated using an online DOGMA tool (Wyman et al. 2004). Finally, The assembled and annotated chloroplast genome was submitted to GenBank database (accession no. MN931250).

The complete cp genome of the *A. villosum* was 164,069 bp, containing a small single copy (SSC) region of 15,353 bp and a large single copy (LSC) region of 88,798bp, which were separated by a pair of inverted repeat (IRs) regions (29,959 bp). The *A. villosum* circular cp genome contained 133 genes, including eight ribosomal RNA genes (4 rRNA species), 38 transfer RNA genes (30 tRNA species) and 87 protein-coding genes (79 PCG species). The most of gene species occurred in a single copy, while 20 gene species occurred in double copies, including four rRNA species (23S, 16S, 5S and 4.5S rRNA), eight tRNA species (trnA-UGC, trnI-CAU, trnI-GAU, trnH-GUG, trnL-CAA, and trnN-GUU, trnR-ACG, trnV-GAC) and eight PCG species (rps7, rps12, rps19, rpl2, rpl23, ycf1, ycf2 and ndhB). Eighteen genes contain intron (12 protein-coding genes and 6 tRNA genes). The overall GC content of the circular genome was 36.05%.

To obtain its evolution status of *A. villosum* within the order Zingiberales, the phylogenetic relationships were constructed by complete chloroplast genomes of 22 species (*Xiphidium caeruleum*, *Arabidopsis thaliana* and *Panax notoginseng* as the outgroup). The alignment was performed using software MAFFT (Katoh and Standley 2013). A maximum likelihood (ML) tree was generated by MEGA6.0 (Tamura et al. 2013) using 1000 bootstrap replicates. ML bootstrap values of all nodes were 100%, 10 Zingiberaceae species form one branch, *A. villosum* is a sister species to *A. krervanh* and *A. compactum* (Figure 1).

**Figure 1.**
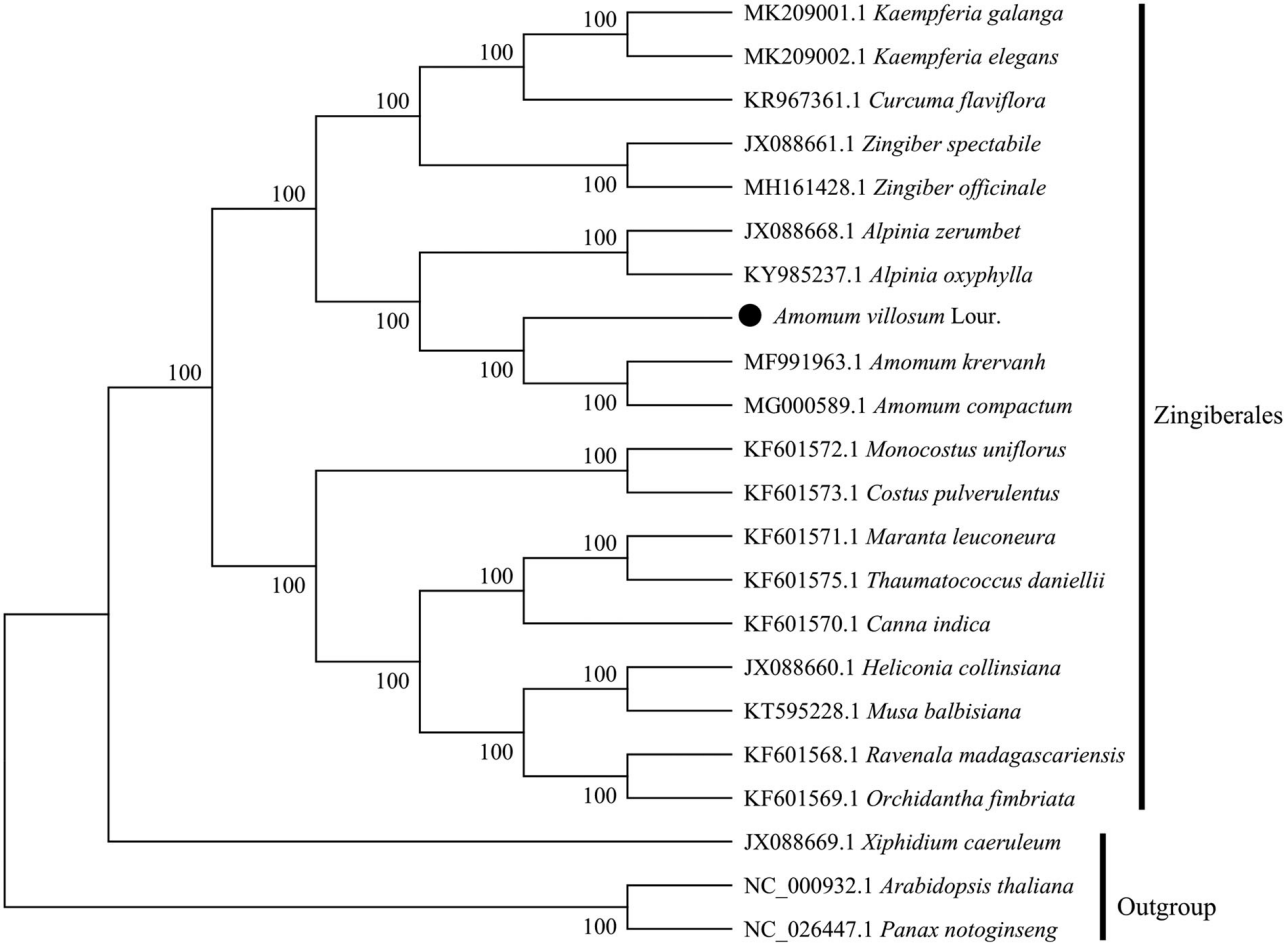
Phylogenetic tree based on the complete chloroplast genome sequences of *A. villosum* and 21 other species (contain 3 outgroup *Xiphidium caeruleum*, *Arabidopsis thaliana* and *Panax notoginseng*). Numbers on the nodes indicate bootstrap values.
